# β-Escin Effectively Modulates HUVECs Proliferation and Tube Formation

**DOI:** 10.3390/molecules23010197

**Published:** 2018-01-17

**Authors:** Lenka Varinská, Lenka Fáber, Martin Kello, Eva Petrovová, Ľudmila Balážová, Peter Solár, Matúš Čoma, Peter Urdzík, Ján Mojžiš, Emil Švajdlenka, Pavel Mučaji, Peter Gál

**Affiliations:** 1 Department of Pharmacology, Faculty of Medicine, Pavol Jozef Šafárik University, 040 11 Košice, Slovakia; lenka.varinska@upjs.sk (L.V.); lenka.faber@yahoo.com (L.F.); martin.kello@upjs.sk (M.K.); coma.matus@gmail.com (M.Č.); jan.mojzis@upjs.sk (J.M.); 2 Department of Biomedical Research, East-Slovak Institute of Cardiovascular Diseases, Inc., 040 11 Košice, Slovakia; 3 Department of Anatomy, Histology and Physiology, University of Veterinary Medicine and Pharmacy, 040 11 Košice, Slovakia; eva.petrovova@uvlf.sk; 4 Department of Pharmacognosy and Botany, University of Veterinary Medicine and Pharmacy, 041 81 Košice, Slovakia; ludmila.balazova@uvlf.sk; 5 Department of Medical Biology, Faculty of Medicine, Pavol Jozef Šafárik University, 040 11 Košice, Slovakia; peter.solar@upjs.sk; 6 Department of Gynaecology and Obstetrics, Faculty of Medicine, Pavol Jozef Šafárik University, 040 11 Košice, Slovakia; peter.urdzik@upjs.sk; 7 Department of Chemical Theory of Drugs, Faculty of Pharmacy, Comenius University, 831 04 Bratislava, Slovakia; emilsva@yahoo.com; 8 Eurofins SK, Testing Laboratory Bratislava, 811 07 Bratislava, Slovakia; 9 Department of Pharmacognosy and Botany, Faculty of Pharmacy, Comenius University, 831 04 Bratislava, Slovakia; mucaji@fpharm.uniba.sk

**Keywords:** β-escin, HUVECs, angiogenesis, bFGF, CAM

## Abstract

In the present study we evaluated the anti-angiogenic activities of β-escin (the major active compound of *Aesculus hippocastanum* L. seeds). Human umbilical-vein endothelial cells (HUVECs) were used as an in vitro model for studying the molecular mechanism underlying the anti-angiogenic effect of β-escin. We investigated the in vitro effects on proliferation, migration, and tube formation of HUVECs and in vivo anti-angiogenic activity was evaluated in a chick chorioallantoic membrane (CAM) angiogenesis assay. Moreover, the effect on gene expressions was determined by the RT2 ProfilerTM human angiogenesis PCR Array. It was found that β-escin exerts inhibitory effect on the basic fibroblast growth factor (bFGF)-induced proliferation, migration and tube formation, as well as CAM angiogenesis in vivo. The inhibition of critical steps of angiogenic process observed with β-escin could be partially explained by suppression of Akt activation in response to bFGF. Moreover, the anti-angiogenic effects of β-escin could also be mediated via inhibition of *EFNB2* and *FGF-1* gene expressions in endothelial cells. In conclusion, β-escin affects endothelial cells as a negative mediator of angiogenesis in vitro and in vivo and may therefore be considered as a promising candidate for further research elucidating its underlying mechanism of action.

## 1. Introduction

Mounting evidence has demonstrated that tumor growth and progression depend on tumor angiogenesis. This fundamental principle states that tumor growth beyond a certain size is strictly dependent on vessel growth and spreading [1]. Considering the importance of vascular growth in tumor progression, the inhibition of angiogenesis has been explored as therapeutic prospect to treat cancer [2]. Several anti-angiogenic agents have been developed to inhibit different stages of angiogenesis in tumor growth processes. In this context, the monoclonal antibody designed against vascular endothelial growth factor-A (VEGF-A) (bevacizumab) was the first US Food and Drug Administration (FDA)-approved anti-angiogenic drug for the treatment of metastatic colorectal cancer [3]. Since then, over 10 therapeutics aimed at inhibiting VEGF activity have been approved for cancer treatment and many more are tested in several clinical trials [4]. Next to VEGF, anti-angiogenic strategies have focused on blocking the angiogenesis by selective small molecule tyrosine kinase inhibitors (TKIs) and FDA has approved over 19 oral TKIs for the treatment of malignancies in hematology/oncology [5].

In anti-angiogenic strategies, natural products are also promising candidates for future cancer treatment [6,7,8] and there are several natural drugs already in development [9,10]. One of such natural product is escin, a mixture of acylated triterpene glycosides isolated from the seeds of horse chestnut (*Aesculus hippocastanum* L.). Escin represents complex mixture of triterpene saponins whose aglycones are derivatives of protoascigenin, acylated by acetic acid at C-22 and by either angelic or tiglic acids at C-21. Three fractions of escin, denoted as crypto-, α-, and β-escin have been described in the literature which can be distinguished by the melting point, specific rotation, haemolytic index and solubility in water. β-Escin is the major active component of extracts from horse chestnut seeds and is the molecular form present in major available pharmaceutical products [11] and exerts anti-inflammatory, anti-edematous [12], vasoconstrictor and vasoprotective effects [13], hypoglycemic [14], anti-obesity [15], and ethanol absorption inhibitory [16] activities. The mechanism of the anti-inflammatory and anti-edematous activity of escin is multidirectional. It was demonstrated that escin counteracted ATP reduction and an increase in the activity of phospolipase A_2_. Moreover, escin reduced neutrophil adhesion and aggregation and restrained hyaluronidase activity [11]. Recently, it was documented that the vascular anti-inflammatory mechanism of β-escin involves disturbances in cholesterol homeostasis leading to cytoskeletal perturbations followed by a decrease of nuclear factor-kappaB (NF-κB) activation [17].

Several studies reported that β-escin and its derivatives are also prime candidates as potential cancer chemotherapeutic agents [18,19,20,21]. Recent studies have shown that β-escin has anti-proliferative effects in different cancer cell lines. For example, escin reduced cell proliferation and induced apoptosis in glioma and lung adenocarcinoma cell lines [22], suppressed the metastatic potential of triple-negative breast cancer cells via inhibiting the epithelial-mesenchymal transition [23] as well as decreased pancreatic cancer cell survival and resulted in the sensitization of pancreatic cancer cells to chemotherapeutic agents [24]. The anti-proliferative, and anti-invasive, activities of escin are mostly mediated via the inhibition of NF-κB and NF-κB-regulated gene products [24,25]. Furthermore, several studies in animals suggest that escin is very well tolerated, no dose-related variations in body weight or organ index were observed [23,26,27,28,29]). Clinically, escin has been proven to be well tolerated and efficient in improving the gastrointestinal motility in patients with colorectal cancer [30] and prolonged progression free survival and overall survival in patients with advanced thyroid cancer [31]. Accordingly, it may be speculated that escin has a potential in the prevention and treatment of cancer.

Other studies have also indicated the potential anti-angiogenic properties of β-escin [32]. However, the exact underlying mechanism of β-escin on human umbilical vein endothelial cells (HUVECs) and its effect in the presence of basic fibroblast growth factor (bFGF) are still poorly understood. For this reason, in the present study we aimed to evaluate its anti-angiogeneic effects and potential mechanism of action under bFGF-stimulated conditions. Furthermore, for the first time the gene profiling of HUVECs following β-escin treatment was performed to complete the panel of experiments.

## 2. Results

### 2.1. Analysis of β-Escin

**Figure 1:**
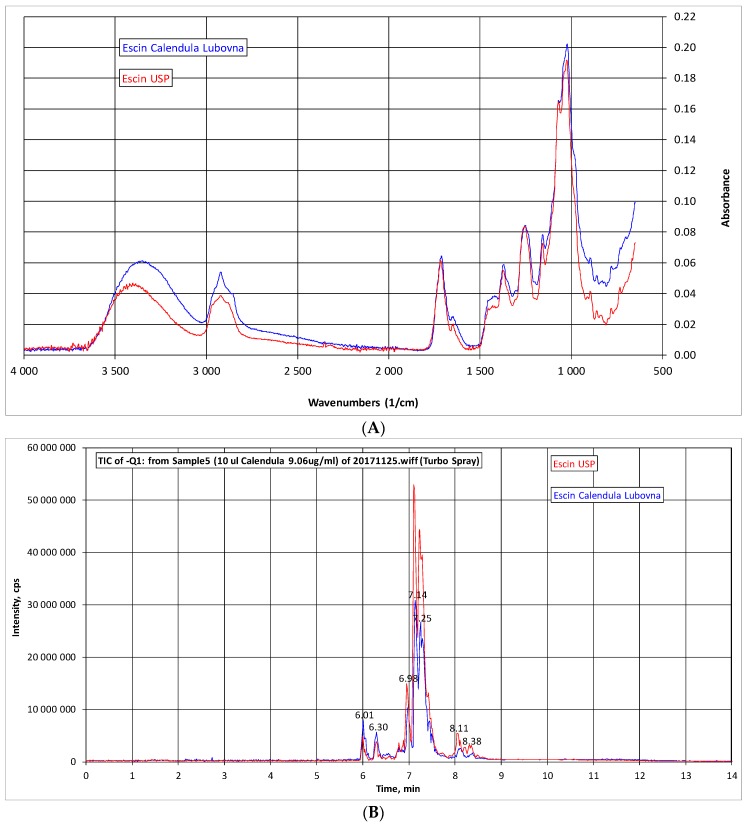
Analysis of β-escin. (**A**) Comparison of IR spectra of β-escin (Calendula, blue) and standard of β-escin (USP, red); (**B**) Comparison of HPLC-MS chromatogram of β-escin (Calendula, blue) and USP standard of β-escin (red).

**Figure 2:**
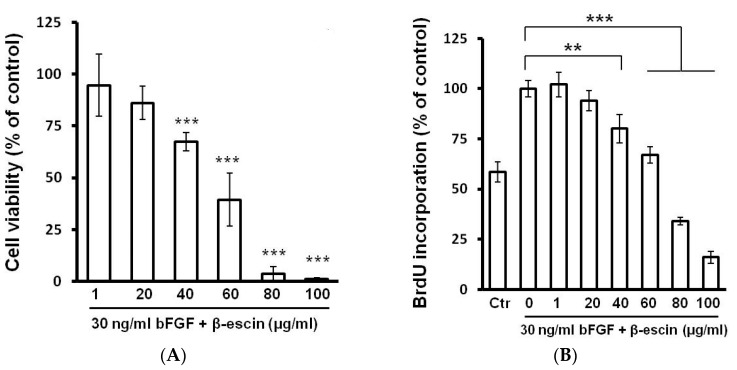
Effect of β-escin on HUVECs. (**A**) Inhibitory effect of β-escin on cell viability. HUVECs were treated with various concentrations of β-escin (1–100 µg/mL) in the presence of bFGF (30 ng/mL) for 48 h. Cell viability was measured by MTT assay as described in the Materials and Methods section. The results represent the mean values ± SD of three independent experiments; (**B**) Proliferation assay using quantitative ELISA analysis of BrdU incorporation into HUVECs during exposure to β-escin (1–100 µg/mL). Data are presented as means ± SD (** *p* < 0.01, *** *p* < 0.001 compared with bFGF treated cells (bFGF control).

In present study tested extract of escin (CALENDULA) was analyzed by IR and HPLC-MS and compared with commercially obtained standard of β-escin (US Pharmacopeial Convention—USP, 99% purity). The IR spectroscopy showed almost identical pattern of both samples (Figure 1A). The HPLC-MS chromatogram exhibited very similar pattern and composition of both samples (Figure 2B). The quantitative analysis revealed that tested extract contains 73.02% of β-escin calculated on dry matter.

### 2.2. Cell Viability, BrdU Incorporation, and Cell Cycle Assay

In order to evaluate the proliferating potential and the cell viability of HUVECs exposed to different concentrations of β-escin (0–100 µg/mL), the MTS reduction assay, 5-bromo-2′- deoxyuridine (BrdU) incorporation assay and cell cycle analysis were carried out. As shown in Figure 2A, basic fibroblast growth factor (bFGF; 30 ng/mL) alone significantly increased cell viability, while the co-incubation with β-escin significantly inhibited bFGF-induced endothelial cell viability at concentrations of 40–100 µg/mL. However, β-escin was not able to significantly reduce the number of HUVECs at the concentrations of 1 and 20 µg/mL. To detect the potential anti-proliferative effect of this compound, the BrdU proliferation assay was used. β-Escin influence on HUVECs proliferation rate was quantified after 48 h of culture, became statistically significant from the concentration of 40 μg/mL for cells treated with β-escin and bFGF (Figure 2B). However, concentrations of 80 μg/mL and 100 μg/mL were found to be toxic, also confirmed by flow cytometric analysis of the cell cycle with the presence of significantly increased sub-G1 population with degraded DNA (data not shown). β-escin at the concentration of 60 μg/mL led to significant changes of cell cycle including reversion of cells back to S phase (potential anti-proliferative effect), but with no increase of sub-G1 population (Table 1). A similar effect was observed at the concentration of 40 μg/mL (data not shown). Of note, low β-escin concentrations (1 and 20 μg/mL) treatment exhibited no significant enrichment in any cell cycle phase compared to the bFGF control (data not shown). In order to work with no toxic and no apoptotic concentration, we selected the IC_50_ at 60 µg/mL of β-escin for subsequent experiments.

### 2.3. β-Escin Inhibits bFGF-Stimulated Migratory Ability Of HUVECs

**Figure 3:**
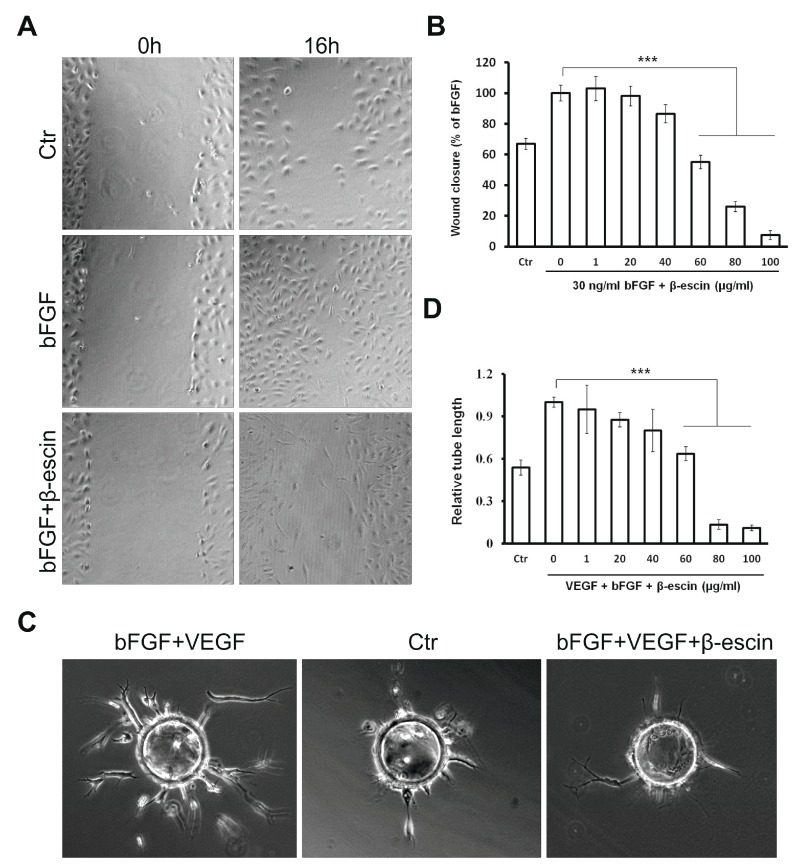
Influence of β-escin on bFGF-induced endothelial cells migration. (**A**) Confluent monolayer of HUVECs was wounded at 0 h. Subsequently, cells were stimulated with 30 ng/mL bFGF in the presence or absence of β-escin at the indicated concentration for 16 h; (**B**) Effect of β-escin (1–100 µg/mL) on bFGF-induced endothelial cells migration. Values are mean ± SD from 2 cultures in 4 independent experiments (*** *p* < 0.001 versus bFGF alone); (**C**) HUVECs exhibit significant reduction of angiogenesis in fibrin gel bead assay after treatment with β-escin (60 µg/mL); (**D**) Effect of β-escin (1–100 µg/mL) on tube-like vessel formation. The quantification shows reduced sprout lengths. For each experiment, three wells for each condition were quantified. Experiments were repeated three separate times, and representative data are shown. Error bars represent ± SD (*** *p* < 0.001 versus bFGF alone).

The migration of endothelial cells is an important step of angiogenesis. A wound healing migration assay was therefore applied to β-escin. As illustrated in Figure 3A,B, the HUVECs migrated into the wounded area actively in control group after 16 h incubation. bFGF at 30 ng/mL significantly facilitated HUVECs migration to the wounded area whereas β-escin significantly inhibited HUVECs migration at the concentration of 60 µg/mL. This effect was not due to cell death, because the HUVECs did not show any morphological changes including membrane blebbing or cell shrinkage suggesting that this compound may inhibit angiogenesis by interfering with normal migratory regulation. Compared to bFGF control, cells treated with 1–40 μg/mL of β-escin did not significantly influence the migration of endothelial cells. Incubation with 80 and 100 μg/mL of β-escin led to the inhibition of bFGF-mediated migration. However, it was caused rather due to the cytotoxic effect of these concentrations (Figure 3B).

### 2.4. Anti-Angiogenic Effects of β-Escin on HUVECs In Vitro and in the CAM Model In Vivo

After 3 days incubation of HUVECs, the control wells (VEGF + bFGF) showed branching pattern of tube-like capillaries. However, tube-like vessels formation by HUVECs was reduced in the wells exposed to β-escin in a concentration-dependent manner with different potencies. A picture of the inhibitory effects induced by 60 µg/mL β-escin on tube-like capillaries formation is illustrated in Figure 3C,D. It was found that the endothelial cells attached to particles had been proliferated and migrated through the fibrin gel in control wells (30 ng/mL bFGF + 25 ng/mL VEGF). However, capillary tube formation was suppressed in wells treated with 60 µg/mL of β-escin. The concentration of 80 and 100 µg/mL showed the highest inhibitory effects on three-dimensional culture of HUVECs but with the cytotoxic effect on cells. Treatment of culture cells with β-escin at the concentrations of 1–40 µg/mL displayed a very limited and no significant effect on the tube-like vessel formation. To confirm the anti-angiogenic effect of β-escin at the concentration of 60 µg/mL, the effect on microvessel formation in vivo was studied in the chick chorioallantoic membrane (CAM) model. The number of vessel branch points was determined in the absence and presence of β-escin.

**Figure 4:**
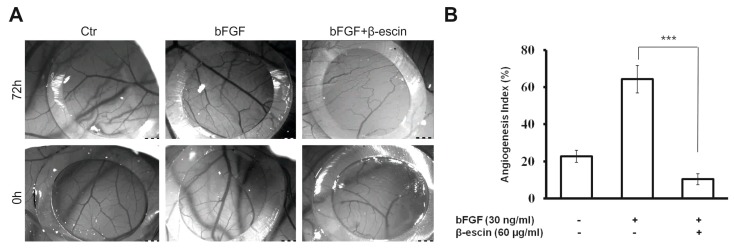
β-Escin reduced capillary formation in vivo. (**A**) Effect of β-escin on angiogenesis in CAM assay. The CAM models were prepared using 7-day-old chick embryos treated as described in Materials and methods. Sterilized silicone ring was positioned on CAM surface and 30 µL of the sample under test was placed within the ring. After incubation for 72 h, CAMs were photographed with a digital camera. Each group contained five CAMs and the experiment was repeated three times; (**B**) The results are summarized in the graph as the angiogenesis index (the mean ± SD of new vessel branch points per field) for each experimental variable. Error bars represent ± SD (*** *p* < 0.001 versus bFGF alone).

As shown in Figure 4A,B, β-escin treatment significantly reduced the blood vessel branch point formation in the chicken embryos when compared with the control (30 ng/mL bFGF). The tested and control groups revealed normal CAM vasculature with no peculiar signs or evidence of vascular trauma. The zero mortality in all groups tested indicates that the dose of the substance tested did present any morphologic signs of toxicity. This result provided evidence that the anti-angiogenic effects of β-escin may be important in its anti-cancer activity.

### 2.5. β-Escin Suppressed bFGF-Downstream Signaling Pathway

To further investigate the underlying mechanism of β-escin’s anti-angiogenic activity, the expression of several proteins involved in regulation of angiogenesis were determined by western blot in HUVECs following β-escin treatment.

**Figure 5:**
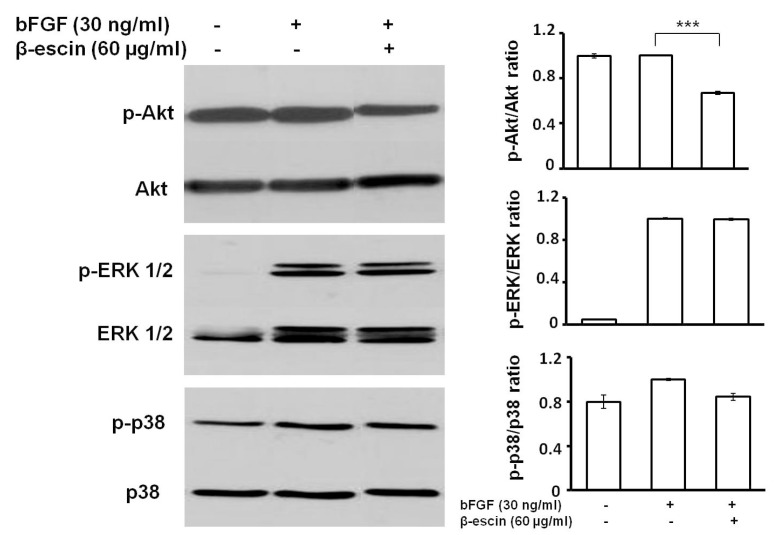
Western blot analysis after β-escin treatment. HUVECs were pre-treated with the indicated concentration of β-escin and then stimulated with 30 ng/mL of bFGF for 30 min before collection. Phosphorylated Akt, ERK 1/2 or p38 were detected by specific antibodies. The pictures shown are representative of three independent experiments. Western blots were quantified by densitometry and the ratio of phosphorylated Akt, phosphorylated ERK 1/2 or phosphorylated p38 to their total counterpart was expressed as mean ± SD of three experiments (*** *p* < 0.001 versus bFGF alone).

As shown in Figure 5, β-escin (60 µg/mL) did not alter bFGF-induced activation of extracellular signal-regulated kinase (ERK) 1/2 in HUVECs and only slightly inhibited the p38 activation. Phosphorylation of Akt, however, was significantly suppressed by β-escin treatment compared to the bFGF-treated cells. In contrast, total levels of Akt, ERK 1/2 and p38 were not affected by β-escin treatment. The above results revealed that β-escin inhibited angiogenesis by directly targeting bFGF-stimulated Akt signaling pathways activation in endothelial cell.

### 2.6. Gene Profiling of HUVECs after β-Escin Treatment

The Human Angiogenesis RT2 Profiler PCR Array profiles the expression of 84 key genes involved in the biological processes of angiogenesis. Quality control parameters included in the Human Angiogenesis RT2 Profiler PCR Array (positive PCR controls and reverse transcription controls) showed a good reproducibility and efficiency based on the web based program of RT2 Profiler PCR Array Data Analysis. Comparison of the various gene expressions of HUVECs after exposure to β-escin (60 µg/mL) in the presence of bFGF and bFGF-treated control is shown in Table 2. Our results showed that several genes of HUVECs that were treated with β-escin were up-regulated compared to control (the *EDN1* gene), while others (the *EFNB2*, *FGF1* and *TIMP3* genes) were down-regulated.

## 3. Discussion

Deregulation of angiogenesis by natural products is being accepted as a good target for cancer prevention and treatment [33]. It was reported that β-escin inhibited tumor growth and development not only by its potent anti-proliferative and apoptotic effects against different cancer cell lines but also by the inhibition of angiogenesis [32]. According to this fact, we provided a thorough insight into the cellular and molecular mechanisms of the anti-angiogenic effect of plant-based β-escin both on HUVECs and using CAM assay. We showed that β-escin could modulate in vitro HUVECs migration and proliferation. Consistent with our results, previous report showed a potent inhibitory effect of β-escin on angiogenesis by depressing proliferation and migration, reaching an inhibition at 40 µg/mL [32]. In our experiments we observed an IC_50_ of β-escin at 60 μg/mL. Our efficient concentration is higher than in the previously published studies [17,32] which may also be related to its composition (73.02% content of β-escin in Calendula samples). However, the inhibitory activity at this concentration did not correlate with the cytotoxicity of the drug. The absence of cytotoxicity was clarified by the prolonged incubation of endothelial monolayers with this concentration. It neither changed the morphology of cells nor induced cell death (data not shown). Moreover, no significant cytotoxic effect was confirmed by flow cytometry analysis. β-Escin treatment at the concentration of 60 μg/mL did not increased sub-G1 population (apoptotic cells) likely making this plant-based compound suitable for further experiments. More importantly, our extract was previously tested on selected cancer cell lines [19] where we clearly demonstrated anti-proliferative concentrations in the IC_50_ ranged from 21.2 µg/mL to 42.9 µg/mL. Hence, we found different sensitivity of cancer and non-cancer cells which might be of clinical importance.

VEGF and bFGF are considered as essential chemotactic factors during angiogenesis which initiate cell migration, proliferation, adhesion, and interaction of endothelial cells to extracellular matrix followed by the tubular network formation [34]. Any agent inhibiting one or more of the above processes can impair angiogenesis and thus, suppresses tumor growth and metastasis. In our study we observed that capillary tube formation was significantly decreased in wells treated with 60 µg/mL of β-escin in vitro under VEGF and bFGF-stimulated conditions. Altogether, β-escin showing inhibition of endothelial cell proliferation, migration, and capillary tube formation emphasize its importance in targeting various key attributes of the angiogenesis process.

In order to determine whether such activity would be applicable to a clinical cancer setting, we also investigated the ability of β-escin, to inhibit angiogenesis in vivo. The CAM assay was carried out to verify the anti-angiogenic activity of tested compound. This assay is frequently used to find out the angiogenic as well as the angiosuppressive potential of various molecules [35]. As expected, we have observed an increase in the vessel numbers and branches in the bFGF control group. However, β-escin treatment has resulted in suppressed normal branching of blood vessels in the developing CAMs. More importantly, this concentration had no harmful effect on preexisting vessels and did not affect the embryo development and survival which correlates with our in vitro findings.

To further investigate the underlying mechanism of the anti-angiogenic properties observed following β-escin treatment, we also examined angiogenesis-related signaling pathways. It is known that the mitogen-activated protein kinases (MAPKs) family including ERK and p38 MAPK and phospatidylinositol-3-kinase (PI3K)/Akt activation are necessary and sufficient to promote angiogenesis [7,36,37], whereas the inhibition of these pathways might result in successful anti-angiogenic and anti-tumor effects [38,39,40]. In this context we have shown that β-escin is able to inhibit Akt phosphorylation, whereas no effect was observed on phosphorylation of ERK and p38. From this point of view, it appears that the anti-angiogenenic activity of this plant-derived compound might be due to the inhibition of bFGF-induced Akt activation.

However, we cannot exclude other underlying mechanisms of action of β-escin. In line with this, expression profiling of HUVECs was performed to assess the regulatory role of β-escin involved in the underlying signaling pathways in angiogenesis. Our results showed that among the 84 tested angiogenesis-related genes, β-escin significantly up-regulated the *EDN1* gene, while the *EFNB2*, *FGF-1* and *TIMP3* genes were down-regulated in HUVECs. In detail, endothelin-1 (a protein encoded by the *EDN1* gene), a potent vasoconstrictor, might be linked with the β-escin-induced vasoconstrictive effects reducing blood flow and in such a way the growth of newly formed tissue. Recent study, by using quantitative global and phosporylated proteomics technological methods, revealed that ephrin-B2 (a protein that in humans is encoded by the *EFNB2* gene) promotes proliferation, survival and migration of HUVECs [41]. Similarly, FGF-1 functions as modifier of endothelial cell migration and proliferation as well as an angiogenic factor [42]. Considering the fact that the proteins encoded by the *EFNB2* and *FGF-1* genes act in multiple ways on stimulating tumor angiogenesis, β-escin may negatively influence angiogenesis by down-regulating these genes. Surprisingly, the compound also down-regulated the *TIMP-3* (Tissue Inhibitor of Metalloproteinase-3) expression. In fact, TIMP-3 inhibits the activities of MMP-1, -2, -3, -9 and -13 [43] as well as inhibits angiogenesis by blocking VEGF binding to VEGFR-2 [44]. Therefore, its down-regulation was rather unexpected and has raised some concerns regarding the safety use of the drug in further clinical trials. Therefore, further studies using animal tumor models are warranted to support clinical usefulness of β-escin in cancer prevention and treatment.

## 4. Materials and Methods

### 4.1. Reagents

Medium 199 (M199) supplemented with 20 mM HEPES (M199), and newborn calf serum (NBCS, heat-inactivated prior use) were obtained from Cambrex (Verviers, Belgium). l-Glutamine, sodium dodecyl sulfate (SDS), and dimethyl sulfoxide (DMSO) were purchased from Sigma-Aldrich (St. Louis, MO, USA). Vascular endothelial growth factor (VEGF)-A was purchased from R&D Systems (Minneapolis, MN, USA); recombinant human fibroblast growth factor basic (FGF-2) was purchased from Thermo Fisher Scientific (Waltham, MA, USA). The tested compound β-escin was a gift from CALENDULA, a.s. (Nová Ľubovňa, Slovak Republic) and dissolved in medium M199. Antibiotics (ATB) penicillin and streptomycin were obtained from Invitrogen (Carlsbad, CA, USA). Human Angiogenesis RT2 Profiler™ RNA RT-PCR Array was purchased from SABiosciences (Qiagen, Valencia, CA, USA). Other materials used in the methods described below are specified in detail in related references or in the text or were purchased from standard commercial sources.

### 4.2. Cell Culture

Human umbilical vein endothelial cells (HUVECs) were isolated, cultured, and characterized as previously described [45,46] following the rules of the Declaration of Helsinki with informed consent of donors and approved by local ethical committee. Cells were cultured on gelatin-coated dishes in cM199 (=M199 medium supplemented with 20% heat-inactivated new born calf serum, 150 µg/mL crude endothelial cell growth factor (ECGF), 5 U/mL heparin, 100 IU/mL penicillin, and 100 µg/mL streptomycin) at 37 °C under 5% CO_2_/95% air atmosphere. Twenty-four hours prior to the experiments the endothelial cell cultures were refreshed with a medium without ECGF and heparin. Cell viability, estimated by trypan blue exclusion, was higher than 95% before each experiment.

### 4.3. IR Spectra and HPLC-MS Analysis

IR spectra: IR spectra were measured on an Impact 400 spectrometer (Nicolet, Madison, WI, USA) using the ATR FTIR (attenuated total reflectance Fourier transform infrared spectroscopy) method with germanium crystal. Samples were examined directly in the solid state.

Quali/quantitative analysis of saponins in the extract was obtained by HPLC-DAD-MS. The measurements were performed with a HPLC chromatograph (Agilent Technologies 1200 Series, Walbron, Germany), equipped with a diode array spectrometer and MS ion trap as detectors (AB SCIEX 3200 Q TRAP LC/MS/MS System, Applied Biosystems, Foster City, CA, USA). MS spectra were recorded in negative Q1 mode in the range of 1065-1175 Da. Parameters: declustering potential −90, entrance potential: −12, curtain gas: 10, ion spray voltage: −4500, temperature 550, ion source gas 1:50, ion source gas 2:50, interface heater: on. An Agilent (Santa Clara, CA, USA) Poroshell 120 EC-C18 (4.6 × 50 mm, 2.7-Micron, P.N. 699975-902) column temperate at 30 °C was used as stationary phase. The mobile phases were solvent A (methanol with 1mM HCOONH_4_ and 1% HCOOH) and solvent B (H_2_O with 1 mM HCOONH_4_ and 1% HCOOH). The elution gradient: 0 min 30% A + 70% B; 7 min. 100% A; 10 min. 100% A; 11 min. 30% A + 70% B; 25 min. 30% A + 70% B. Flow: 0.3 mL/min.

Identification of constituents was achieved on the basis of MSn fragmentation experiments and comparison of the obtained fragmentation pathways with reference compound of escin (β-escin) of the USP (US Pharmacopeial Convention, 99% purity), batch No. R023G0. Quantification of saponins was obtained with the method of calibration curve: β-escin (USP) was used for quantification of saponins in the sample at six different concentrations (data not shown).

### 4.4. MTS Cell Viability Assay

Cell viability and proliferation were determined using colorimetric microculture assay with MTS (3-(4,5-dimethylthiazol-2-yl)-5-(3-carboxymethoxyphenyl)-2-(4-sulfophenyl)-2*H*-tetrazolium) dye (Promega, Madison, WI, USA). Cells were seeded at a density of 4 × 103 cells/well in 96-well polystyrene microplates. Twenty-four hours after cell seeding, different concentrations (1, 20, 40, 60, 80 and 100 μg/mL) of the compound in the presence of 30 ng/mL of recombinant bFGF and 25 ng/mL of recombinant VEGF [47] were tested. After 48 h of incubation, 10 μL of MTS were added to the each well. After an additional 3 h, cell proliferation was evaluated by measuring the absorbance at wavelength 490 nm using the automated Cytation™ 3 Cell Imaging Multi-Mode Reader (Biotek, Winooski, VT, USA). Absorbance of control wells was taken as 100%, and the results were expressed as a percentage of the untreated control.

### 4.5. 5-Bromo-2′-deoxyuridine (BrdU) Cell Proliferation Assay

Cell proliferation activity was directly monitored by quantification BrdU incorporated into the genomic DNA during cell growth. DNA synthesis was assessed using colorimetric cell proliferation ELISA assay (Roche Diagnostics GmbH, Mannheim, Germany) following the vendor’s protocol. Briefly, 4 × 103 cells/well in 80 µL medium were plated in a 96-well tissue culture grade flat bottom plate. The next day, cells were treated with or without the studied compound β-escin (1–100 µg/mL) in the presence of 30 ng/mL of recombinant bFGF for 48 h. After 24 h of treatment, cells were incubated with BrdU labeling solution for another 24 h at 37 °C followed by fixation and incubation with anti-BrdU peroxidase conjugate for an additional 1.5 h at room temperature. Finally, after substrate reaction, color intensity was measured with an automated Cytation™ 3 Cell Imaging Multi-Mode Reader at 450 nm (reference wavelength: 690 nm).

### 4.6. Analysis of Cell Cycle Distribution 

For flow cytometric analysis of the cell cycle, floating and adherent cells were harvested together 48 h after treatment, washed in cold PBS, fixed in cold 70% ethanol and kept at −20 °C overnight. Prior to analysis, cells were washed twice in PBS, resuspended in staining solution (final concentration 0.1% Triton X-100, 0.5 mg/mL ribonuclease A and 0.025 mg/mL propidium iodide-PI), incubated in the dark at RT for 30 minutes and analyzed using a FACS Calibur flow cytometer (Becton Dickinson, San Jose, CA, USA).

### 4.7. Assessment of Monolayer Integrity

Endothelial cells were seeded and grown to confluence in 6-wells plates and then treated with β-escin (60 µg/mL) in cM199 medium. Pictures of the monolayers were taken using an inverted light microscope (Olympus IX70, Tokyo, Japan) equipped with cooled CCD camera (Hamamatsu ORCA-Flash4.0 LT Digital CMOS camera C11440-42U, Hamamatsu, Japan) at the time points 24 h, 48 h and 72 h and the number of cells were counted.

### 4.8. Two-Dimensional Migration (Wound Healing) Assay

The motility of HUVECs was assayed using a wound healing assay [48]. Briefly, endothelial cells were cultured on a 24-well plate in the cM199 medium until confluent. A 2 mm pipette tip was used to wound the monolayer of cells. Afterwards, the medium was replaced with fresh ECGF and heparin-free medium containing the studied compound at different concentrations in the presence of 30 ng/mL recombinant bFGF. The wounded area was photographed at the start (t = 0 h) and at a specific time point (t = 16 h). The migration distance (gap size) was determined using image analysis software. The experiments were performed in duplicate wells and repeated three times with cells from different donors.

### 4.9. Fibrin Gel Bead Assay

The effect of β-escin on sprouting efficiency of HUVECs was assessed in the in vitro fibrin gel bead assay as described previously [49]. Briefly, 2500 Cytodex beads (GE Healthcare, Chicago, IL, USA) were incubated with 106 HUVECs (5 h, 37 °C, and 5% CO_2_) and plated overnight on a 10 cm dish to remove unattached cells. Next day, the cell-covered beads were resuspended to a concentration of ~350/mL in 3 mg/mL fibrinogen (Sigma-Aldrich) solution containing 0.15 U/mL aprotinin (Sigma-Aldrich), 30 ng/mL bFGF, and 25 ng/mL VEGF. Aliquots were mixed with thrombin (Sigma; 0.625 U/mL), distributed in 12-well plates (150 mL/well), and left to clot for 5–10 min. After further incubation (10 min, 37 °C, and 5% CO_2_), medium containing 30 ng/mL bFGF and 25 ng/mL VEGF was added to cover the clot. Sprout formation was examined microscopically and photographs were taken using an Olympus IX70 inverted light microscope equipped with a cooled CCD camera (Hamamatsu ORCA-Flash4.0 LT Digital CMOS camera C11440-42U) and analyzed using ImageJ network analysis software (Bethesda, MD, USA).

### 4.10. Western Blot Analysis

Protein lysates from our cells were prepared using Laemli lysis buffer containing 1 mol/L Tris/HCl (pH 6.8), glycerol, 20% SDS (sodium dodecyl sulfate) and deionized H_2_O and a sonication process. Protein concentration was evaluated using the Pierce^®^ BCA Protein Assay Kit (Thermo Scientific, Rockford, IL, USA) and measured using an automated Cytation™ 3 Cell Imaging Multi-Mode Reader (Biotek) at wavelength 570 nm. Equal amounts of protein samples were separated on 12% SDS-polyacrylamide gel and electrophoretically transferred (200 mA, 2 h) onto a PVDF Blotting Membrane (GE Healthcare, Chicago, IL, USA). Afterwards the membrane was blocked for 1 h using 4% non-fat dry milk. The following primary antibodies were used: anti-phospho-ERK 1/2 (Cell Signaling Technology, Beverly, MA, USA, 1:2000), anti-phospho-Akt (Cell Signaling Technology, 1:2000) and anti-phospho-p38 (Cell Signaling Technology, 1:1000). The membranes were incubated with the indicated antibodies in 5% BSA in TBS-Tween at 4 °C during the night. The next day, the membranes were washed in TBS-Tween (3 × 5 min) and incubated with a corresponding secondary antibody (goat-anti-rabbit-HRP (Santa-Cruz Biotechnology, Santa Cruz, CA, USA, 1:2000) and goat-anti-mouse-HRP (Dako, Carpinteria, CA, USA; 1:2000) for 1 h at room temperature, respectively. After incubation, the membranes were again washed in TBS-Tween (3 × 5 min), and protein was visualized by enhanced chemiluminescence (Thermo Scientific, Rockford, IL, USA) according to the manufacturer’s instructions on X-ray film (Pierce, Rockford, IL, USA). Signal intensity of p-ERK, p-Akt and p-p38 was determined densitometrically (software Quantity One, Bio-Rad Laboratories, Hercules, CA, USA) and expressed relative to total ERK, Akt or p38.

### 4.11. RNA Isolation And cDNA Synthesis

After treatment of HUVECs with β-escin (60 µg/mL) in the presence or absence of bFGF, the total cellular RNA was isolated from the cells by a Qiagen RNeasy^®^ Mini Kit (Catalog # 74104) according to the manufacturer’s instructions. RNA samples underwent DNase treatment and removal. RNA quantification was performed with spectrophotometry (ND-1000; NanoDrop Products, Thermo Fisher Scientific, Wilmington, DE, USA), after which 250 ng of total RNA was analyzed by agarose gel electrophoresis to confirm integrity. The resultant RNA was stored at −80 °C. Only samples pure enough (A260/A230 ratio > 1.8, A260/A280 ratio = 1.8–2.0), with reasonable concentration (>100 ng/μL), were used as templates for cDNA synthesis. First-strand complementary DNA was synthesized from total RNA (0.5 μg) using the RT2 First-Strand Kit (Catalog # 330401, Qiagen, Germany). In brief, 0.5 μg of total RNA was added to 2 μL of Buffer GE (5× gDNA Elimination Buffer), and the final volume was made up to 10 μL with RNase-free water. The mixture was denatured at 42 °C for 5 min and then immediately cooled by placing on ice for 1 min. Reverse transcription was performed after adding 10 μL of reverse transcription mix to the solution. The reaction mixture was incubated at 42 °C for 15 min, after which it was terminated by heating at 95 °C for 5 min. The cDNA samples generated were then diluted with 91 μL RNase-free water and stored at −20 °C until further analysis.

### 4.12. Gene Expression Profiling

Real-time PCR was carried out by using a Biorad CFX96 (Bio-Rad Laboratories). Gene expression was examined using the Human Angiogenesis RT2 Profiler™ PCR Array (cat. # 330231D, Qiagen). The RT2 Profiler™ PCR Array contains built-in primers for 84 tested and 5 housekeeping genes and positive control elements to determine the efficiency of the reverse transcription reaction, performance of the PCR reaction, and detection of genomic DNA contamination. The PCR mixture for 96 reactions contained 1350 μL of RT2 SYBR Green Mastermix (Qiagen), 102 μL cDNA template, and 1248 μL RNase-free water. The PCR reaction mix was added to the wells of the PCR plate in equal amounts (25 μL), and then the real-time PCR cycling program was run. The thermal cycling program recommended by the plates manufacturer for the Biorad CFX96 was as follows: 10 min at 95 °C followed by 40 cycles: denaturation at 95 °C for 15 s, with 60 s annealing and elongation at 60 °C, followed by melting curve analysis.

### 4.13. PCR Data Analysis and Statistics

RT profiler data were analyzed using SABiosciences data analysis software (http://pcrdataanalysis.sabiosciences.com/pcr/arrayanalysis.php). The ΔΔ*C*_t_ method was used for data analysis. Specifically, fold-changes for each gene were calculated as difference in gene expression between β-escin exposure in the presence or absence of growth factor. A positive value indicated gene up-regulation and a negative value indicated gene down-regulation. Each experiment was independently repeated at least twice (as recommended by the manufacturer’s guidelines for statistical significance). Genes with greater than 2.0 fold change in expression compared to control were identified as significant (*p* < 0.05).

### 4.14. Chicken Embryo Chorioallantoic Membrane (CAM) Assay

Fertilized chicken eggs (20 pcs) were received from a certified farm (Parovske Haje, Slovak Republic) and incubated with storage blunt end up in a forced-draft incubator at 37.5 °C, approximately 60–65% humidity after cleaning with 70% ethanol. On ED 3 (embryonic day 3) of incubation period, 2 mL of albumen was aspirated with a syringe needle (20 G) so as to detach the developing CAM from the top part of the shell. On ED 7, a window of around 1.5–2.0 cm^2^ was gently opened with a serrated scissors on the blunt end of the egg without damaging the embryo. Sterilized silicone ring (inner diameter—6 mm) was positioned on CAM surface avoiding major blood vessels and 30 µL of the sample under test was placed within the ring. After 72 h, the vascularization of CAM was evaluated. The photographs of CAM blood vessels formation inside of rings were obtained using a Olympus SZ61 stereomicroscope and ARTCAM-300MI digital camera (both Tokyo, Japan). The experiments were repeated three times with 5 eggs per group.

### 4.15. Statistical Analysis

Results are expressed as mean ± SD (standard deviation). Statistical analyses of the data were performed using standard procedures, with one-way ANOVA followed by the Bonferroni Multiple Comparisons Test. Differences were considered significant when P values were less than 0.05.

## 5. Conclusions

Overall, the present study shows that β-escin is a potent and promising anti-angiogenic agent that inhibits multifunctional events of bFGF-induced angiogenesis both in vitro and in vivo. β-escin treatment caused the repression of Akt activation in the HUVECs in response to bFGF, indicating that this signaling pathway could be major target in the molecular mechanism of β-escin. To the authors’ knowledge, this is the first study demonstrating that the anti-angiogenic effects of β-escin could be also mediated via inhibition of expression of *EFNB2* and *FGF-1* genes in endothelial cells. Of note, this is also the first study demonstrating its tricky effect on TIMP-3 expression. However, the question whether here observed micromolar concentrations of escin, a complex mixture of molecules, many of which still remain undefined, will ever find a place in modern medicine remains open.

## Figures and Tables

**Table 1:** The cell cycle distribution in HUVEC cells after 48 h treatment with β-escin (c = 60 μg/mL) was assessed by flow cytometry. Data are expressed as means ± SD of three independent experiments.

Treatment	Control	bFGF	bFGF + β-Escin 60 μg/mL
Sub-G_1_	2.45 ± 0.05	3.20 ± 0.70	3.80 ± 1.06
G_0_/G_1_	72.67 ± 3.77	40.05 ± 1.15 **	42.60 ± 1.38 **
S	10.73 ± 2.07	18.15 ± 1.45 *	38.65 ± 2.99 **^,++^
G_2_/M	14.15 ± 1.75	38.60 ± 1.90 **	14.95 ± 1.95 ^++^

The significant differences between control and β-escin-treated cells were signed as * *p* < 0.05, ** *p* < 0.01; β-escin-treated cells vs. bFGF, ^++^ *p* < 0.01.

**Table 2:** Fold change of gene expression in HUVECs exposed to β-escin (60 µg/mL) in the presence of bFGF compared to bFGF-treated control.

Gene Symbol	Gene Name	β-Escin/Control
*EDN1*	Endothelin-1	+2.3
*EFNB2*	Ephrin B2	−2.3
*FGF1*	Fibroblast Growth Factor 1	−5.3
*TIMP3*	Tissue Inhibitor of Metalloproteinases 3	−4.2
